# Electrospun Polycaprolactone/Aloe Vera_Chitosan Nanofibrous Asymmetric Membranes Aimed for Wound Healing Applications

**DOI:** 10.3390/polym9050183

**Published:** 2017-05-21

**Authors:** Sónia P. Miguel, Maximiano P. Ribeiro, Paula Coutinho, Ilídio J. Correia

**Affiliations:** 1 CICS-UBI—Centro de Investigação em Ciências da Saúde, Universidade da Beira Interior, Av. Infante D. Henrique, 6200-506 Covilhã, Portugal; soniapmiguel@gmail.com (S.P.M.); maxprarib@gmail.com (M.P.R.); coutinho.ipg@gmail.com (P.C.); 2 UDI-IPG—Unidade de Investigação para o Desenvolvimento do Interior, Instituto Politécnico da Guarda, 6300-559 Guarda, Portugal

**Keywords:** Aloe Vera, asymmetric membranes, chitosan, electrospinning, wound dressings

## Abstract

Today, none of the wound dressings available on the market is fully capable of reproducing all the features of native skin. Herein, an asymmetric electrospun membrane was produced to mimic both layers of skin. It comprises a top dense layer (manufactured with polycaprolactone) that was designed to provide mechanical support to the wound and a bottom porous layer (composed of chitosan and Aloe Vera) aimed to improve the bactericidal activity of the membrane and ultimately the healing process. The results obtained revealed that the produced asymmetric membranes displayed a porosity, wettability, as well as mechanical properties similar to those presented by the native skin. Fibroblast cells were able to adhere, spread, and proliferate on the surface of the membranes and the intrinsic structure of the two layers of the membrane is capable of avoiding the invasion of microorganisms while conferring bioactive properties. Such data reveals the potential of these asymmetric membranes, in the near future, to be applied as wound dressings.

## 1. Introduction

Skin structure and functions are often compromised by traumatic events (e.g., thermal burns, cuts, lacerations or surgical incisions) or chronic wounds (e.g., pressure ulcers or diabetic foot ulcers) [1,2,3,4]. When a large area of skin is lost, the immediate coverage of the wound is mandatory for avoiding water/blood losses, for preventing bacterial invasion, as well as decreasing the pain felt by patients [5].

Currently, autologous skin grafting remains the “gold standard” treatment used in the clinic. However, this approach cannot always be used due to the extent of the wound, limited availability of donor sites, scar formation, slow healing, and patient morbidity. Allogeneic and xenogeneic skin grafts are alternative therapeutic approaches that have been also used, although their application has associated risks of immune rejection, disease transmission, and limited availability of skin bank collection [6,7]. To overcome these limitations, researchers have developed different skin substitutes, some of them used in the clinic. Nevertheless, none of these skin substitutes is fully capable of reestablishing the native structure and functions of skin [8,9].

Among the skin substitutes developed so far, asymmetric membranes to be applied as wound dressings systems [10,11,12,13] have captured the attention of researchers. The top layer of these membranes can be designed to avoid bacterial invasion and also prevent wound surface dehydration, whereas the underlying layer can be conceived to remove the wound exudate and to promote cell infiltration and proliferation [8].

Heretofore, dry/wet and scCO_2_-induced phase inversion methods have been the most used to produce asymmetric membranes [14,15]. In recent times, electrospinning emerged as an alternative to produce bilayer membranes aimed to improve the healing process [16]. To produce electrospun membranes, a charged polymer solution is initially accelerated under a high-voltage electrostatic field, and subsequently, the fiber jet travels through the atmosphere to allow solvent evaporation, leading to the deposition of solid polymer fibers on the collector. The produced fibers display a high surface to volume ratio and porosity, properties that are crucial for increasing cell adhesion, growth, migration, differentiation, and angiogenesis [17,18,19].

Herein, an asymmetric membrane, composed of two distinct layers (aimed to reproduce the epidermis and dermis layers of skin), was produced through electrospinning. A synthetic polymer, polycaprolactone (PCL), was used to produce the top layer that is aimed to act as a protective barrier [13,20]. On the other hand, Chitosan (CS) and Aloe Vera (AV) were used to manufacture the bottom layer. CS is a natural polysaccharide, known for its capacity of promoting collagen synthesis and also by exhibiting bactericidal and hemostatic properties. Such features triggered its use in the production of wound dressings, namely in HidroKi^®^, Patch^®^, Chitopack^®^, Tegasorb^®^, and KytoCel^®^ [16,21,22,23,24,25]. Although, limited solubility of CS at physiological pH and high viscosity in acid solutions hinder its application in electrospinning. To overcome such a drawback, synthetic polymers such as Poly(ethylene oxide) (PEO), Polyvinyl alcohol (PVA), Poly(lactide-co-glycolide) (PLGA) have been blended with this polymer to enable the production of CS nanofibers [26,27,28,29,30]. AV is a member of the *Liliaceae* family and it has been widely used for the treatment of different skin disorders, like burns, infections, and other dermatologic conditions [31]. The mucilaginous gel, present in AV leaves, has a higher water content (~99%), which is fundamental for wound hydration. Furthermore, different compounds like amino acids, salicylic acid, ascorbic acid, vitamin A, and vitamin E are also found in AV. Such biomolecules are responsible for conferring antibacterial, anti-inflammatory, and antioxidant properties to AV [31,32,33]. In addition, AV is also known for, promoting fibroblast proliferation, increasing collagen synthesis, and ultimately, enhancing the wound healing process [31,32,33,34,35,36,37].

## 2. Experimental Section

### 2.1. Materials

AV leaves were obtained from 5-year-old plants (*Aloe barbadensis Miller*) that were bought from a Portuguese botanic shop. 3,3,3 Trifluoroethanol (TFE) was purchased from Acros Organics (Jersey City, NJ, USA). Lysozyme from chicken egg was acquired from Alfa Aesar (Haverhill, MA, USA). Fetal bovine serum (FBS) (free from any antibiotic) was purchased from Biochrom AG (Berlin, Germany). Glacial acetic acid (AA) was obtained from LabChem (New York, NY, USA). Paraformaldehyde (PFA) was obtained from Merck, SA (Algés, Portugal). Normal Human Dermal Fibroblasts (NHDF) cells were purchased from PromoCell (Labclinics, S.A., Barcelona, Spain). 3-(4,5-Dimethylthiazol-2-yl)-5-(3-carboxymethoxyphenyl)-2 (4-sulfophenyl)-2H-tetrazolium (MTS) was bought from Promega (Madison, WI, USA). Amphotericin B, CS (low molecular weight (LMW: 50,000–190,000 Da), Dulbecco’s modified Eagle’s medium (DMEM-F12), Ethylenediaminetetraacetic acid (EDTA), Gentamicin, Glutaraldehyde, Kanamycin, LB Broth, Phosphate-buffered saline solution (PBS), PCL (80,000 Da), PEO, Sodium hydroxide (NaOH) and Trypsin were acquired from Sigma-Aldrich (Sintra, Portugal). Quant-iT Pico Green dsDNA assay kit was obtained from ThermoFisher Scientific (Waltham, MA, USA). Bovine serum albumin was obtained from VWR International (Carnaxide, Portugal). *Staphylococcus aureus* clinical isolate (*S. aureus*) ATCC 25923 and *Escherichia Coli DH5α* (*E. coli*) were used as models of prokaryotic organisms to evaluate the bactericidal activity of the membranes.

### 2.2. Methods

#### 2.2.1. Extraction of the AV Gel

The extraction of AV gel was performed according to a method previously described elsewhere [34,35]. Briefly, AV leaves were initially washed with distilled water to remove any dirt from their surface and, subsequently, the skin of the leaf was carefully separated from the parenchyma using a scalpel-shaped knife (Thermo Fisher Scientific, Waltham, MA, USA). The samples obtained were then homogenized in a blender (Sigma-Aldrich, Sintra, Portugal) and then filtered. After, the AV gel was stabilized at 65 °C for 15 min and stored at 4 °C for later use.

#### 2.2.2. Deacetylation of Chitosan

CS was purified and deacetylated through a method previously described in the literature [21]. Briefly, 500 mg of CS LMW was dispersed in 10 mL of NaOH (1M) solution, under magnetic stirring for 4 h, at 50 °C. Then, the mixture was filtered using a Whatman^®^ quantitative filter grade 541:0.22 μm (Sigma-Aldrich, St. Louis, MO, USA) and a Buchner funnel to recover the deacetylated CS. The remaining material was washed extensively until the sample pH reached 7.4. Afterward, the samples were dried at 40 °C overnight. The degree of deacetylation (DD) of CS was determined by using the first derivative UV-spectroscopy (1DUVS) method [38]. UV–Vis CS spectra were acquired in a Thermo Scientific Evolution 201 UV–Vis spectrophotometer (Thermo Fisher Scientific, Waltham, MA, USA).

#### 2.2.3. Production of the Electrospun Asymmetric Membranes

A conventional electrospinning apparatus adapted by us, comprising a high voltage source (Spellman CZE1000R, 0–30 kV) obtained from (Spellman, Corporate Headquarters USA) a precision syringe pump (KDS-100) (acquired from Sigma-Aldrich, Sintra, Portugal), a plastic syringe with a stainless-steel needle (21 Gauge), and an aluminum disk connected to a copper collector, was used to produce the asymmetric membranes.

The top layer of the membrane was produced with a PCL solution (7% PCL (*w*/*v*) in 80% TFE: 20% H_2_O (*v*/*v*)), placed in the syringe and then electrospun at a constant flow rate of 2.5 mL/h, using a working distance of 15 cm and an applied voltage of 25 kV. After, the top layer was produced, 10 mL of CS_PEO solution—composed of 7% CS (*w*/*v*) and 8% PEO (*w*/*v*) (2:1 volume ratio) or CS_AV_PEO ((2:2:1 volume ratio), where an extract of 40% AV gel (*v*/*v*) was used)—was electrospun on top of the PCL electrospun membrane at a constant flow rate of 4.0 mL/h, using a working distance of 12 cm and an applied voltage of 28 kV. The produced asymmetric membranes (PCL/CS_PEO and PCL/CS_AV_PEO) were then characterized through in vitro assays to evaluate their suitability to be used as wound dressings.

#### 2.2.4. Attenuated Total Reflectance–Fourier Transform Infrared Spectroscopy Analysis

The final composition of the electrospun membranes was determined by ATR-FTIR analysis. The membranes’ spectra were acquired with an average of 128 scans, a spectral width ranging from 4000 and 400 cm^−1^ and a spectral resolution of 4 cm^−1^, using a Nicolet iS10 FTIR spectrophotometer (Thermo Scientific, Waltham, MA, USA). Furthermore, the spectra of the raw materials used to produce the membranes were also acquired for comparative purposes.

#### 2.2.5. Characterization of the Mechanical Properties of the Membranes

The mechanical properties of PCL/CS_PEO and PCL/CS_AV_PEO membranes were determined with a Shimadzu AG-X Tensile Testing Machine (Tokyo, Japan) operated at room temperature (RT), under wet and dry conditions, accordingly to the guidelines established by the Standard Test Method for Tensile Properties of Polymer Matrix Composite Materials (ASTM standard D3039/ D3039 M) [15]. The tested samples (*n* = 5) had a width of 2 cm, a gauge length of 6 cm and a thickness ranging from 0.41 to 0.52 mm. The length between the clamps was set to 2 cm and the speed of testing was set to 2 mm/min. In wet conditions, the membranes were immersed in a PBS solution, over 24 h at 37 °C. Load-extension data was recorded and the stress–strain curve of the membranes was determined through Equations (1) and (2), respectively:

(1) $$\mathrm{Stress}=\sigma =\frac{F}{A}$$

(2) $$\mathrm{Strain}=\varepsilon =\frac{\Delta l}{L}$$

where *F* is the applied force; *A* is the cross-sectional area; Δ*l* is the change in length, and *L* is the length between the clamps.

#### 2.2.6. Evaluation of the Porosity of the Produced Membranes

The microporosity of the membranes was determined through a liquid displacement method [25]. Briefly, three specimens were weighed and then immersed in absolute EtOH for 1 h and later on reweighed. The membrane’s porosity was determined through Equation (3):

(3) $$\mathrm{Porosity}\;\left(\%\right)=\frac{W_{s}-W_{d}}{D_{\mathrm{ethanol}}\;\times \;V_{\mathrm{membrane}}}\;\times 100$$

where *W_d_* is the initial weight of dry membrane and *W_s_* is the weight of the swollen membrane, *D*_ethanol_ is the density of the ethanol at room temperature and *V*_membrane_ is the volume of the swollen membrane. For each sample, three replicates were used and the presented data represents the average of the results obtained for each sample.

#### 2.2.7. Determination of Contact Angle at the Surface of the Produced Membranes

The contact angles at the surface of the samples were determined with a Data Physics Contact Angle System OCAH 200 apparatus (DataPhysics Instruments GmbH, Filderstadt, Germany) operating in static mode, at 25 °C and using water as a reference fluid. For each sample, water drops (4 µL) were placed on the surface of the membranes. The reported contact angles are the average of at least three independent measurements.

#### 2.2.8. Water Vapor Transmission Rate (WVTR)

The water vapor diffusion through the PCL/CS_PEO and PCL/CS_AV_PEO membranes was evaluated accordingly to a method previously described elswhere [25]. Briefly, the membranes were used to seal the opening of a glass test tube (1.77 cm^2^) containing 10 mL of ultrapure water to avoid its evaporation. A parafilm tape was used to attach the membrane to the glass tube. Afterward, the membranes were incubated at 37 °C and at specific time points, the amount of water evaporation was obtained by determining the weight loss. The WVTR was estimated through Equation (4):

(4) $$\mathrm{Water}\;\mathrm{Vapor}\;\mathrm{Transmission}\;\mathrm{Rate}=\frac{W_{\mathrm{loss}}}{A}$$

where W_loss_ is the daily weight loss of water and *A* is the area of the tube opening.

#### 2.2.9. Swelling and Enzymatic Degradation

Swelling and degradation tests were performed by immersing all samples in PBS (pH = 5) and PBS containing 13.6 mg/mL lysozyme, at 37 °C, under stirring (60 rpm) for 30 days [35]. All experiments were conducted in triplicate and the solutions were changed periodically in order to guarantee that the enzyme remained active through the study. The swollen sample weights were measured after removing the water excess present at the surface of the membranes, by gently tapping the surface with filter paper. Water uptake was determined through Equation (5).

(5) $$\mathrm{Swelling}\;\mathrm{ratio}\;\left(Q\right)=\frac{W_{t}}{W_{0}}$$

where *W_t_* is the final weight and *W*_0_ is the initial weight of the membranes.

The degradation profile of the samples was determined at specific time points (1, 3, 7, 14, and 30 days). To do that, samples were removed from the solution (PBS with 13.6 mg/mL lysozyme) and weighed, after being completely dried [38]. The degradation percentage at each time point was calculated according to Equation (6):

(6) $$\mathrm{Weight}\;\mathrm{loss}\;\left(\%\right)=\frac{W_{i}-W_{t}}{W_{t}}\;\times \;100$$

where *W_i_* corresponds to the initial weight of the sample and *W_t_* to the weight of the sample at time t.

#### 2.2.10. Protein Adsorption

To characterize protein adsorption at the surface of the membranes, Bovine Serum Albumin (BSA) was used, following a procedure described elsewhere [39]. The membranes were initially placed in a 24-well cell culture plate and 300 µL of BSA (1 mg/mL protein/phosphate buffer) was added to the surface of the electrospun membranes. The plate was then placed in a humidified incubator at 37 °C at specific time points. Empty wells (tissue culture polystyrene (TCPS)) were used as background reference. The non-adherent BSA was removed from wells by washing them with PBS solution. Subsequently, 300 µL of 2% sodium dodecyl sulfate was added to each well and then incubated overnight to extract adhered proteins. The concentration of the adhered proteins was determined through the micro bicinchoninic acid (BCA Kit) assay.

### 2.3. Characterization of the Biological Properties of the Produced Membranes

#### 2.3.1. Characterization of Cell Viability and Proliferation in Contact with the Membranes

The cytotoxic profile of produced membranes was evaluated in vitro following ISO 10993-5. Prior to cell seeding, membranes were placed into 96-well plates and then sterilized, by UV irradiation (254 nm, ~7 mW cm^−2^), over 1 h. NHDF cells were used as model and seeded at a density of 10 × 10^3^ cells per well in the presence of the membranes. Then, the plate was incubated at 37 °C, in a 5% CO_2_ humidified atmosphere, and the culture medium was changed every two days until the end of the assay. After 1, 3, and 7 days of incubation, a MTS assay was performed to characterize the membranes biocompatibility. Briefly, the medium of each well was removed and replaced by a mixture of 100 µL of fresh culture medium and 20 µL of MTS/PMS (phenazine methosulfate) reagent solution. Following this, cells were incubated for 4 h, at 37 °C, in a 5% CO_2_ atmosphere. The absorbance of each sample (*n* = 5) was determined at 492 nm using a microplate reader (Biorad xMark microplate spectrophotometer). Cells cultured without materials were used as a negative control (K^–^), whereas cells cultured with EtOH (96%) were used as positive control (K^+^).

#### 2.3.2. dsDNA Quantification

To assess the cells proliferation rate, the total DNA content was measured with a Quan-iT PicoGreen dsDNA Assay kit, following a protocol previously described in the literature [35,40]. Briefly, NHDF cells were seeded, at a density of 10 × 10^3^ cells/mL, in contact with the produced membranes. After 1, 3, and 7 days, the membranes were washed twice with PBS and then each membrane was transferred into a 1.5 mL Eppendorfs containing 1mL of ultra-pure water. Thereafter, the cell–membrane complexes were subjected to a freeze–thaw cycle and sonicated for 15 min, to lyse the cells. Samples and standards were prepared and mixed with a PicoGreen solution at a 200:1 ratio and placed in a 96-well plate (*n* = 3) [35]. The plate was incubated for 10 min in the dark and fluorescence was measured in a microplate reader using excitation and emission wavelengths of 480 nm and 520 nm, respectively. A calibration curve was performed and samples dsDNA concentrations were determined.

#### 2.3.3. Characterization of the Antimicrobial Properties of the Membranes

##### Analysis of Bacterial Penetration through the Top Layer of the Produced Membranes

*S. aureus* and *E. coli*, gram-positive and gram-negative bacteria, were used to characterize the capacity of the membrane’s top layer to avoid bacterial infiltration within their structure. To accomplish this, transwell systems (Corning Incorporated, New York, NY, USA) were modified with a PCL membrane or a filter paper (0.22 µm) (control group) to act as the interface between the upper and lower chamber. The PCL membrane and the filter paper were inoculated with a bacterial suspension (1 × 10^8^ colony forming units (CFU)/mL), during 24 h, at 37 °C [41]. After, the optical density of the medium culture present in the lower chamber was determined. Posteriorly, the number of colonies that cross the PCL membrane/filter paper were also counted. Furthermore, the PCL membrane and filter paper were recovered and submitted to SEM analysis to evaluate the presence of the bacterial colonies on the upper and lower side of the samples.

##### Characterization of Bactericidal Activity of the Bottom Layers (CS_PEO and CS_AV_PEO) Membranes

The bactericidal activity of the PCL/CS_PEO and PCL/CS_AV_PEO membranes was also characterized using *S. aureus* and *E. coli* as bacteria models. Briefly, each membrane was added to 10 mL of LB broth at pH 6.2, containing 1 × 10^5^ colony forming units (CFU)/mL of early mid-log phase culture and then were incubated at 37 °C, for 24 h. After the incubation period, serial dilutions were prepared and 100 µL of bacterial samples were transferred into LB agar plates. Following overnight incubation at 37 °C, bacterial colonies were counted and expressed as the number of colonies forming units per mL [25,42,43]. The bacterial growth inhibition was calculated through Equation (7):

(7) $$\mathrm{Antibacterial}\;\mathrm{efficiency}\;\left(\%\right)=\;\frac{N_{0\;\;}-N}{N_{0}}\;\times 100$$

where *N*_0_ and *N* each represents the bacteria number of control and experimental group.

To further characterize the bactericidal activity of the membranes, biofilm formation on the membrane’s surface was also studied. To accomplish this, the produced membranes were placed on the surface of a plate of LB agar in contact with *S. aureus* and *E. coli* (1 × 10^8^ colony forming units (CFU)/mL) and then incubated for 24 h, at 37 °C [21]. Following this, biofilm formation at the surface of the membranes was assessed through SEM analysis.

#### 2.3.4. Characterization of the Morphologic Features and Biological Performance of the Electrospun Membranes by SEM Analysis

Biological samples, containing cells or bacteria, were fixed for 4h with 2.5% (*v*/*v*) glutaraldehyde. Following this, samples were washed three times with PBS and then dehydrated with growing concentrations of EtOH (70%, 80%, 90%, and 100%) or freeze-dried for 3 h. Finally, all biological and non-biological samples were mounted onto aluminum stubs with Araldite glue and sputter-coated with gold using a Quorum Q150R ES sputter coater (Quorum Technologies Ltd, Laughton, East Sussex, UK). SEM images were then acquired with different magnifications, using an acceleration voltage of 20 kV, in a Hitachi S-3400N Scanning Electron Microscope (Hitachi, Tokyo, Japan).

#### 2.3.5. Confocal Microscopy Analysis

Confocal laser scanning microscopy (CLSM, Zeiss, Oberkochen, Germany) was used to characterize cell distribution and proliferation at the surface of both layers of the produced asymmetric membranes. Cells (10 × 10^3^ cells/mL) were seeded on the surface of CS_PEO and CS_AV_PEO layers, which were previously placed in µ-Slide 8 well Ibidi imaging plates (Ibidi GmbH, Planegg/Martinsried, Germany). After 1 and 3 days, cells were fixed with 4% paraformaldehyde (PFA) in PBS for 20 min and then stained with the WGA-Alexa 594^®^ conjugate. Cells were then rinsed several times with PBS and labeled with Hoechst 33,342^®^ nuclear probe (2 µM). The 3D reconstruction and image analysis were performed using Zeiss Zen 2010 software (Zeiss, Oberkochen, Germany).

### 2.4. Statistical Analysis

The statistical analysis of the results obtained was performed using one-way analysis of variance (ANOVA), with the Newman-Keuls post hoc test. A *p* value lower than 0.05 (*p* < 0.05) was considered statistically significant.

## 3. Results and Discussion

### 3.1. Characterization of the Morphology of the Membranes

When asymmetric membranes are aimed to be used as wound dressings, they are conceived with a dense top layer, that can protect the wound from physical damage and infection, and a porous and hydrophilic inner layer capable of absorbing the wound exudate and providing a 3D architecture that enables cell adhesion and proliferation [44].

**Figure 1:**
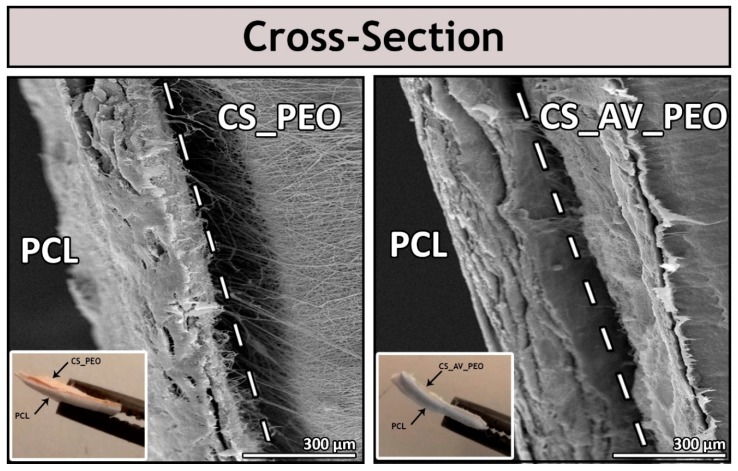
Scanning electron microscopy (SEM) and macroscopic images of the cross-section of the produced bilayer membranes (PCL/CS_PEO and PCL/CS_AV_PEO).

Up to now, different techniques have been used to produce these membranes, including supercritical carbon dioxide (scCO2)-assisted phase, dry/wet-phase inversion, and electrospinning [10,45]. In this study, asymmetric membranes were produced with an electrospinning apparatus. In Figure 1, a denser top layer can be observed that was manufactured with PCL (an FDA-approved biomaterial that has a low cost, is biocompatible, and displays good mechanical properties) [16,46,47,48]. This polymer confers to the top layer suitable mechanical properties, waterproof capacity, avoids bacteria penetration, and regulates the gaseous exchanges between the wound and the surrounding environment. On the other hand, the porous bottom layer was composed either of CS_PEO or CS_AV_PEO and was designed to be able to absorb the wound exudates, promote nutrient exchange, support cell proliferation, and also to confer antimicrobial properties to the membrane. The higher porosity of the bottom layers of the asymmetric membranes was also confirmed by the results obtained in porosity assays (see further details in Section 3.4).

Regarding the macroscopic images (insets of Figure 1), it is possible to identify the two different layers of asymmetric membrane. Such a feature will make easier the future application of the membranes at the wound site.

**Figure 2:**
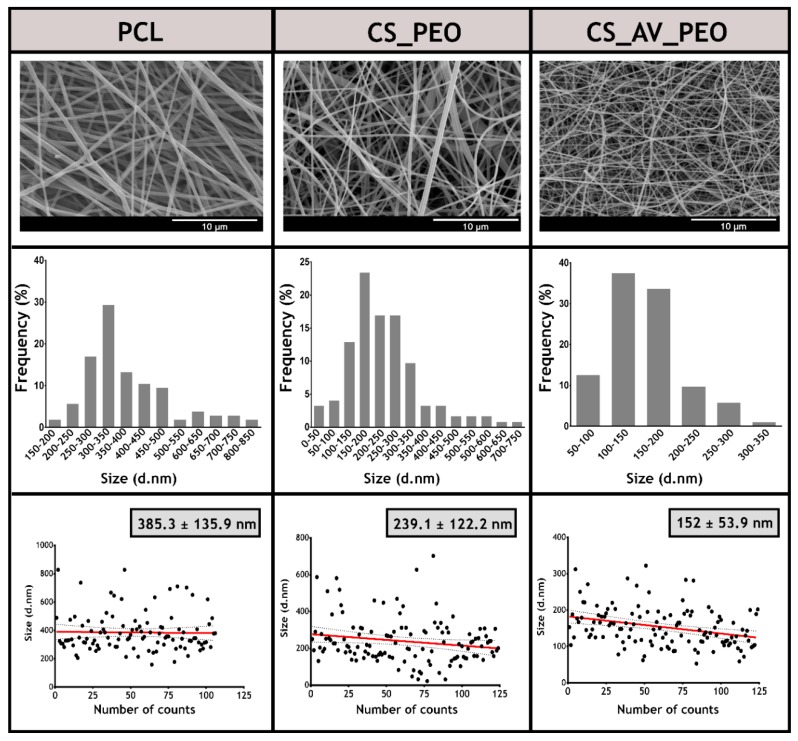
Characterization of the morphologic features of the produced asymmetric membranes. SEM images, fiber diameters distribution and the diameters of different fibers of the top layer (PCL) and bottom layers (CS_PEO or CS_AV_PEO) of the produced bilayer membranes are presented.

The diameter of the electrospun nanofibers present in the membranes was determined through SEM analysis (Figure 2). The top layer displays randomly oriented fibers with average diameters of 385 ± 134 nm. Such a result is in agreement with the data available in the literature for membranes produced with PCL [49]. In turn, the bottom layer of membranes presented a mean diameter of 239 ± 122 nm and 152 ± 54 nm for CS_PEO and CS_AV_PEO, respectively (Figure 2). It seems that the incorporation of the AV gel leads to the production of thinner fibers, due to the reduction of the viscosity of the solution used for nanofiber production. Suganya and their collaborators had already reported a similar effect on the diameter of PCL nanofibers when AV was added to the PCL solution. Nanofibers with a diameter of the 215 ± 63 nm were produced when AV and PCL were blended [36]. On the other hand, Karuppuswamy et al. previously obtained PCL nanofibers with diameters of 346 ± 63 nm and PCL/AV nanofibers with diameters of 307 ± 42 nm [50].

Moreover, the diameters of nanofibers produced herein are within the size range displayed by collagen fibers present in natural ECM (50–400 nm). The increased roughness presented by the produced membranes leads to a higher surface area available for cell adhesion. In addition, the high porosity exhibited by the nanofibers is fundamental in preventing fluid accumulation, enhance oxygen permeation, and ultimately improve cell adhesion and proliferation [51,52,53].

### 3.2. Attenuated Total Reflectance-Fourier Transform Infrared Spectroscopic Analysis

**Figure 3:**
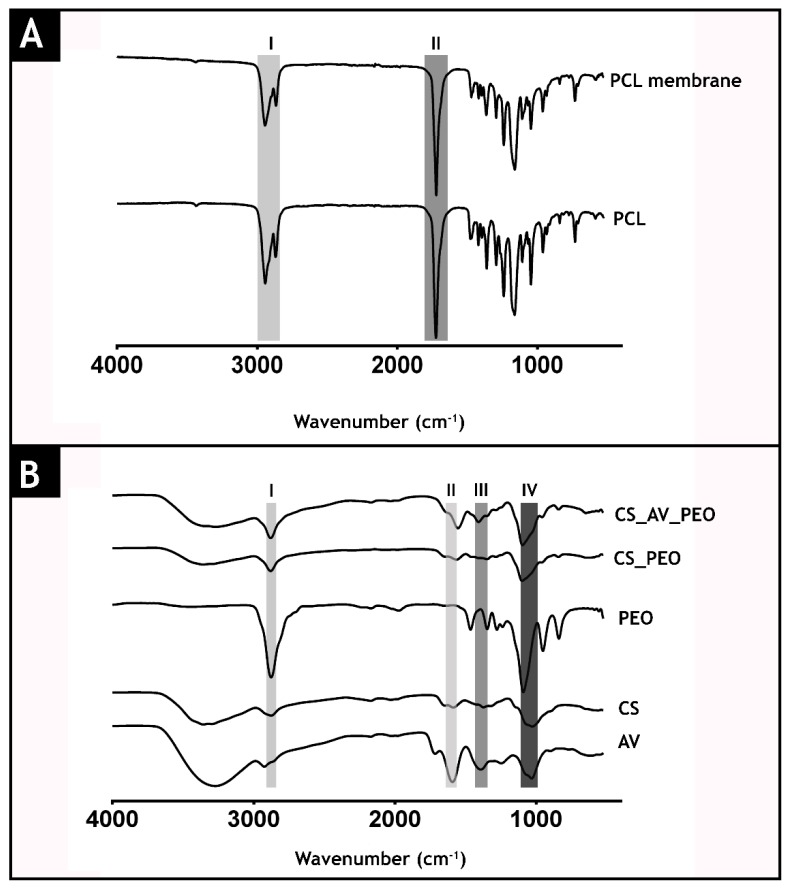
Attenuated total reflectance-Fourier transform infrared spectroscopic analysis (ATR-FTIR) of the produced membranes and of their raw materials: top layer (**A**) and bottom layers (**B**).

The acquired ATR-FTIR spectra of raw materials and of the produced membranes are presented in Figure 3. The spectrum of the top layer (Figure 3A) presents its characteristic peaks at 2942 cm^−1^ (I) (asymmetric CH_2_ stretching), 2868 cm^−1^ (symmetric CH_2_ stretching), 1723 cm^−1^ (II) (carbonyl stretching), 1292 cm^−1^ (C-O and C-C stretching), 1239 cm^−1^ (asymmetric C-O-C stretching) and 1161 cm^−1^ (symmetric C-O-C stretching) [54,55].

In Figure 3B, it is possible observe the characteristic bands of PEO in the region between 2900 cm^−1^ and 2850 cm^−1^ (CH_2_ (band I)) [56]. Furthermore, the spectrum of the CS_AV_PEO layer (Figure 3B) displays the characteristic peaks of AV at 1714 (O-acetyl esters (band II)), 1592 (asymmetrical COO^−^ stretching (band III)), 1246 (glucan units), 1030 (glycosidic bond), and 606 cm^−1^ (C–H ring vibration). Additionally, the bottom layer also exhibits peaks at 2876 cm^−1^ (aliphatic C–H stretch), 1586 cm^−1^ (NH_2_ stretch), 1372 cm^−1^ (–C–O stretching of the primary alcohol group) and at 1028 cm^−1^ (C–O–C glycosidic bond (band IV)) that belong to CS [57,58].

### 3.3. Characterization of Mechanical Properties of the Membranes

Membranes to be used as wound dressings must present mechanical properties that can support angiogenesis, the lymphatic system, nerve bundles, and other structures found in native skin [59,60,61]. Herein, the mechanical properties of the produced membranes were evaluated in dry and wet conditions [62].

**Figure 4:**
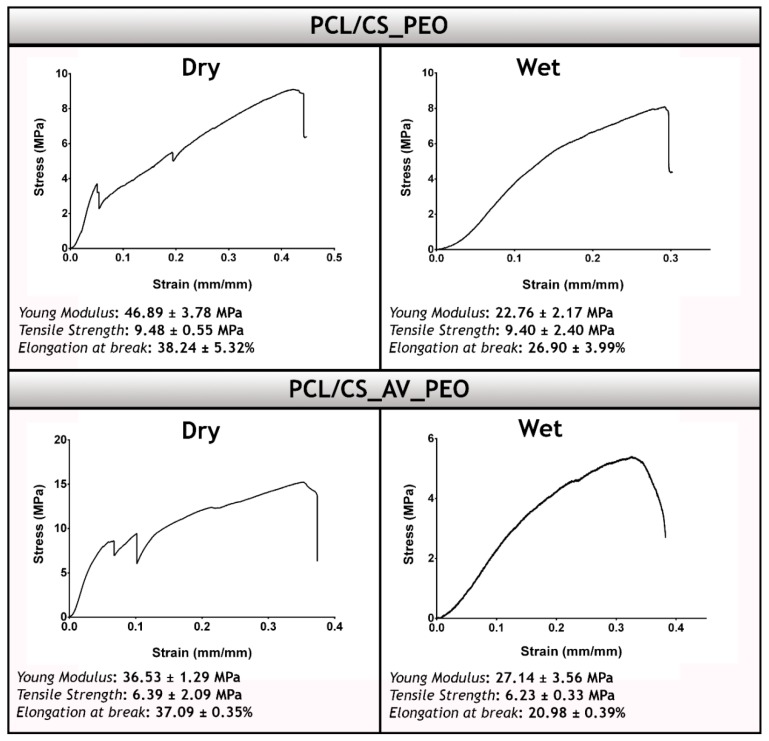
Typical stress-strain curves and mechanical properties determined for the produced membranes in dry and wet conditions. Young´s modulus, tensile strength, and elongation at break of PCL/CS_PEO and PCL/CS_AV_PEO membranes.

Through the analysis of the stress-strains curves (Figure 4), it is possible to notice that the membranes display a two stage fracture in the dry state. Such a result is explained by the presence of natural polymers (CS and AV) in the bottom layer that have weaker mechanical properties. In the wet state the membranes do not show the two stage break, since CS and AV are less brittle in wet conditions [15,63].

PCL/CS_PEO membranes presented a Young Modulus of 46.89 ± 3.78 MPa in dry state, whereas in wet conditions a value of 22.76 ± 2.17 MPa was obtained (Figure 4). Such increase in the elasticity was also observed for PCL/CS_AV_PEO membranes, i.e., 36.53 ± 1.29 MPa and 27.14 ± 3.56 MPa for the dry and wet state, respectively. Moreover, in the wet state, the PCL/CS_PEO and PCL/CS_AV_PEO membranes displayed tensile strengths of 9.40 ± 2.40 MPa and 6.23 ± 0.33 MPa, respectively. On the other hand, in the dry state, the PCL/CS_PEO and PCL/CS_AV_PEO membranes presented tensile strengths of 9.48 ± 0.55 MPa and 6.39 ± 2.09 MPa, respectively. The elongation-to-break assays revealed that PCL/CS_PEO and PCL/CS_AV_PEO membranes can bear a strain of 26.90 ± 3.99% and 20.98 ± 0.39% for the wet state and 38.24 ± 5.32% and 37.09 ± 0.35% for the dry state, correspondingly. Despite the samples presenting a different behavior in dry and wet conditions, the produced membranes show a Young modulus, tensile strengths, and elongation-to-break values that are very close to that displayed by native skin (Young modulus (4.6–20.0 MPa), tensile strengths (5.00–30.00 MPa) and elongation-to-break (35.00–115.00%)).

This emphasizes the suitability of the produced membranes to confer adequate mechanical support during the tissue remodeling process, as well as, avoiding possible side effects resulting from the stress-shielding mechanism [60,64]. The excellent mechanical performance of the produced membranes can be explained by the presence of PCL within the top layer. In the literature, it has been widely described that this synthetic polymer is capable of providing enhanced mechanical properties due to its chemical and thermal stability [65,66].

### 3.4. Characterization of Membrane Porosity

**Figure 5:**
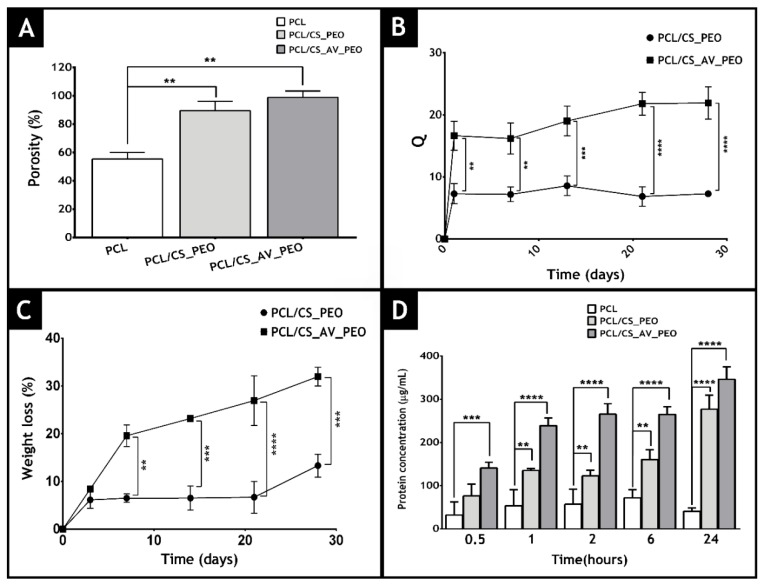
Characterization of the total porosity (**A**), swelling profile (**B**), weight loss (**C**), and protein adsorption (**D**) on the membrane’s surface at different time points.

In the area of tissue engineering, the porosity of a material is essential to provide void spaces for cell accommodation, migration and also for the exchange of nutrients between the 3D construct and the surrounding environment [50]. Herein, a liquid displacement method was used to determine membrane porosity (ethanol was used as displacement fluid) [67]. The data obtained is presented in Figure 5A and reveals that top layer (PCL) displays the lowest porosity (55 ± 5%), which is essential for avoiding microorganism penetration [15,41]. On the other hand, the bottom layers, composed of CS_PEO or CS_AV_PEO, showed porosities of 89.5 ± 5.3% and 97.8 ± 4.5%, respectively. Such results can be explained by the higher number of spaces available between the CS_AV_PEO nanofibers, which have a lower diameter.

Several researchers have previously highlighted that materials with porosities above 90% are the most appropriate for skin tissue engineering applications since they are able to provide the required space for cell accommodation, migration, nutrient exchange, and production of a new ECM [46,68,69,70,71,72].

### 3.5. Membrane Surface Wettability

Material surface wettability is a crucial parameter that affects cell adhesion, proliferation, and differentiation [73]. The surface wettability is usually characterized by determining the water contact angle (WCA) [74,75,76]. In the literature, it is described that cells are more prone to adhere, spread, and grow on moderate hydrophilic substrates (40° < WCA < 70°) than on hydrophobic (WCA > 90°) or very hydrophilic ones (WCA < 20°) [21,77,78].

In the present study, the membranes’ top layer exhibited a hydrophobic character (WCA of 126.2° ± 1.21°), due to the presence of aliphatic polyester PCL [79,80]. Conversely, CS_PEO and CS_AV_PEO layers presented WCA values of 75.58° ± 12.57° and 69.06° ± 3.78° which are characteristic of moderate hydrophilic materials. Such WCA values can be explained by the presence of natural polymers (CS and AV) that have functional groups like amides, esters, and hydroxyl groups which confer hydrophilic character to the bottom layer [81,82]. During the wound healing process, hydrophilic materials are able to provide moist environments, that enhance the healing process [83].

### 3.6. Water Vapor Transmission Rate

As described above, an ideal wound dressing besides providing a moist environment, must also avoid wound dehydration, as well as be capable of removing the wound exudate [15,21,43]. An accumulation of exudate at the wound site leads to the breakdown of extracellular matrix components or to the maceration of the healthy surrounding tissues, that ultimately induces more pain to the patient [84].

Furthermore, it has been reported that wound dressings with WVTRs values ranging from 2000–2500 g/m^2^/day provide an adequate level of moisture and prevent exudate accumulation [85]. Here, the produced membranes showed similar WVTR values: The PCL/CS_PEO membrane displayed a WVTR of 1452.61 ± 86.08 g/m^2^/day, while the PCL/CS_AV_PEO had a value of 1252.35 ± 21.22 g/m^2^/day. Although the determined WVTR values are outside the range of that displayed by an ideal dressing, there are several commercially available wound dressings that have WVTR values outside this range, like Tegaderm (3M, Maplewood, MN, USA), Bioclusive (Johnson-Johnson, New Brunswick, NJ, USA), and Op Site (Smith & Nephew, London, UK), which have WVTRs of 491 ± 44, 394 ± 12, and 792 ± 32 g/m^2^/day, respectively [86,87,88,89].

### 3.7. Characterization of the Membranes’ Swelling Profile

The characterization of the swelling profile of the membranes is fundamental to evaluate the capacity of these membranes to absorb the wound exudate. To accomplish that, membranes were incubated in a PBS solution and their weight was monitored at specific timepoints. The obtained results showed that the PCL/CS_AV_PEO membrane displays a higher water absorption ratio (~20) than the PCL/CS_PEO membrane (~10) (Figure 5B). Such a result is explained by the incorporation of hydrophilic materials in the composition of membranes [21,31,34,35]. Furthermore, the swelling profile displayed by the membranes is compatible with the removal of the excess of exudate from the wound, which is usually produced during the inflammatory phase of the healing process (1–3 days after the injury occurs). Based on this data, the PCL/CS_AV_PEO membranes are the ones more appropriate to absorb any excess of exudate that may be present in the wound.

### 3.8. Characterization of Degradation Profile of the Membranes

Commercially available wound dressings are usually non-degradable and they need to be removed from the wound. Such removal can affect the healing process since it can induce the formation of scar tissue as well as increase the risk of bacterial contamination [78,90]. To overcome this drawback, researchers started to produce biodegradable wound dressings. However, their rate of degradation must be proportional to the rate of healing or regeneration of the compromised tissue [91,92]. Herein, the degradation profile of the developed membranes was studied using lysozyme, an enzyme found in human serum, which is capable of degrading CS-based materials in vivo [93,94]. The results obtained revealed that after 30 days of incubation, the PCL/CS_AV_PEO and PCL/CS_PEO membranes had weight losses of ~30% and ~10%, respectively (Figure 5C). Such variation in weight is explained by AV capacity to reduce the number of interactions occurring between CS molecules, thus facilitating the enzymatic degradation [35].

Despite PCL displaying a slow degradation profile in vitro [79], it is expected that under in vivo conditions it degrades faster. A variety of enzymes, like matrix metalloproteinase (MMP-1), gelatinase-A (MMP-2), and stromelysin-1 (MMP-3) are secreted by macrophages, epidermal cells, and fibroblasts during the wound healing process and may contribute to the enzymatic breakdown of PCL [95,96,97].

### 3.9. Evaluation of Protein Adsorption on the Membranes’ Surface

When a wound dressing is placed at the wound site, it is exposed to body fluids and almost immediately proteins present in these fluids become adsorbed onto the dressing’s surface. Protein adsorption can have a direct impact on the material’s biocompatibility since it can influence cellular attachment [76,98,99]. Indeed, cells adhere to proteins adsorbed on material surfaces through membrane receptors that recognize specific amino acid sequences present in adhesive proteins (e.g., fibronectin, and vitronectin) [100]. Herein, to characterize protein adsorption to the PCL, PCL/CS_PEO and PCL/CS_AV_PEO membranes´ surface, albumin was used as a model protein (Figure 5D). Albumin is the most abundant protein in serum and after an injury occurs this protein is accumulated at the wound site, during the early phase of the healing process. Subsequently, the absorbed albumin is replaced by fibronectin and vitronectin [76]. The results obtained revealed that albumin did not become adsorbed onto the PCL membrane (Figure 5D), as previously reported in the literature [101,102]. Conversely, the concentration of protein adsorbed onto PCL/CS_PEO and PCL/CS_AV_PEO membranes increases with time and no statistically significant difference was noticed between these samples. Such a finding was expected since both membranes exhibit rough, porous and hydrophilic surfaces (WCA < 90°). Furthermore, other authors claim that the protein adsorption at the membranes’ surface can occur through the interaction of proteins with the amine groups of CS, which have a high affinity for negatively charged groups found on proteins and cell membranes [103,104,105].

### 3.10. Evaluation of Cell Viability and Proliferation in the Presence of Membranes

The wound healing process is known by its complexity and interaction of different cell types with matrix components that act together to re-establish the 3D structure and the functions of the damaged tissue. Herein, NHDF cells, enrolled in the synthesis of collagen, fibronectin, and other biomolecules were used to evaluate the cell response to the presence of the developed membranes [106].

**Figure 6:**
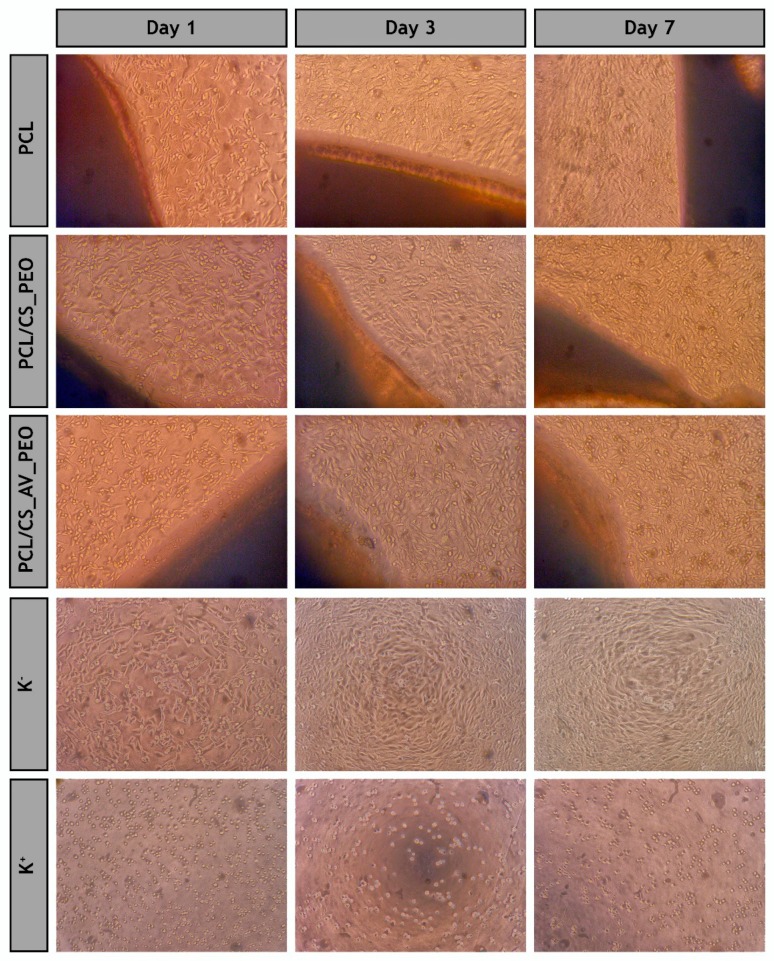
Optical microscopic images of Normal Human Dermal Fibroblast (NHDF) cells in presence of the different produced membranes (PCL, PCL/CS_PEO and PCL/CS_AV_PEO) after 1, 3, and 7 days of incubation; K^−^ (negative control); K^+^ (positive control).

Optical microscopic images of the NHDF cells in contact with membranes after 1, 3, and 7 days are presented in Figure 6. These images show that NHDF cells did not suffer any morphological variation when in contact with the membranes, displaying a similar shape to those of K^−^, where cells were incubated with culture medium.

**Figure 7:**
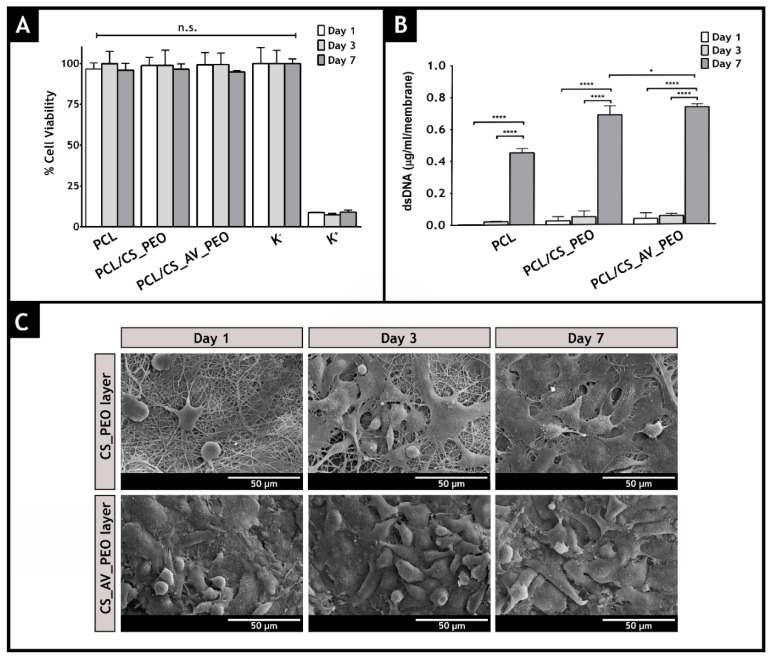
Characterization of the biological performance of the produced membranes. NHDF cell viability was evaluated when the cells were incubated with produced membranes (**A**) and the content of dsDNA of NHDF cells adhered to the surface of the developed membranes was determined after 1, 3, and 7 days of incubation. The statistical analysis of the results was performed using one-way ANOVA with Newman–Keuls test (**B**); SEM micrographs of NHDF cells morphology at the surface of the different electrospun membranes after 1, 3, and 7 days are presented in (**C**).

Moreover, the cytotoxic profile of the membranes was evaluated through an MTS assay, over 1, 3, and 7 days (Figure 7A). The results revealed that all membranes did not induce any cytotoxic effect on NHDFs, over 7 days. Furthermore, the dsDNA quantification results (Figure 7B) are compliant with the data obtained in the MTS assay, suggesting that NHDFs remain viable and proliferate over at least 7 days, when they are seeded in contact with the produced membranes.

Besides being biocompatible, a wound dressing must also promote cell attachment, growth, and proliferation. Herein, the cell adhesion to the surface of the membranes was characterized by SEM analysis (Figure 7C). The obtained SEM images evidenced the bioadhesive character of the bottom layer of the produced membranes.

**Figure 8:**
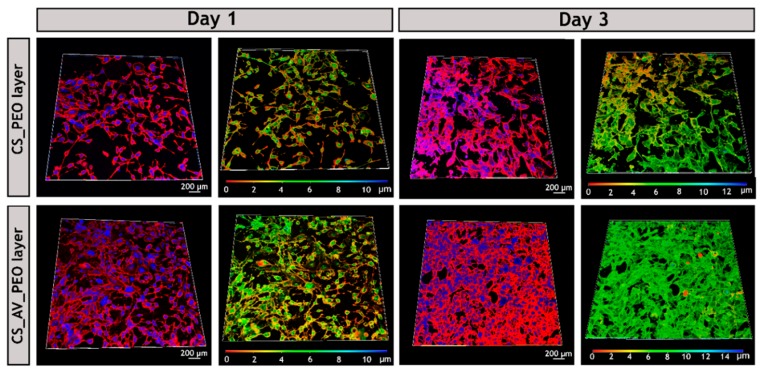
Confocal laser scanning microscopy (CLSM) images of fibroblast cells cultured on the surface of CS_PEO and CS_AV_PEO layers and color coded depth analysis (red 0 µm, blue 10–14 µm) after 1 and 3 days. Blue channel: cell nuclei labeled Hoechst 33342^®^; red channel: cytoplasm stained with WGA-Alexa 594^®^ conjugate.

After 7 days of incubation, it was possible to visualize that cells adhere and proliferate on the nanofibrous network, which is crucial for tissue regeneration [107]. Additionally, CLSM analysis was also performed and the acquired images (Figure 8) demonstrate that, after 3 days of incubation, the fibroblast cells have a higher attachment, proliferation, and growth when they are in contact with the PCL/CS_AV_PEO membranes, in comparison to the PCL/CS_PEO ones. Similar results have been already reported for other materials that have AV in their composition [108]. The exact mechanisms through which AV stimulates cell proliferation and growth factor production in fibroblasts are still unknown. Previous studies speculated that AV interacts with the growth factor receptors of the fibroblasts and stimulates cell activity and proliferation [36,109]. Such interaction occurs between the active components, such as mannose-6-phosphate and/or acemannan, present in AV and the mannose receptor available on the fibroblast’s surface [110,111]. The results obtained through the color coded depth analysis of the membranes showed that fibroblasts migrate to the interior of the membrane, with some cells being observed between 8–10 µm within the polymeric structure, after 3 days of incubation (Figure 8). Such data emphasize that the pores available on the bottom layers allow cellular internalization, and also provide an effective nutrient supply and metabolic waste removal, thus leading to the restoring of the structure and functions of the native tissue [21,112].

### 3.11. Characterization of the Antimicrobial Properties of the Produced Membranes

Skin injuries are prone to microorganism contamination, that can often interrupt the healing process and lead to life-threatening complications. Such are the demands for wound dressings capable of avoiding bacterial invasion and growth. In this study, the antimicrobial properties of the produced membranes were characterized by using *S. aureus* (gram-positive bacterium) and *E. coli* (gram-negative bacterium) as models [113,114].

**Figure 9:**
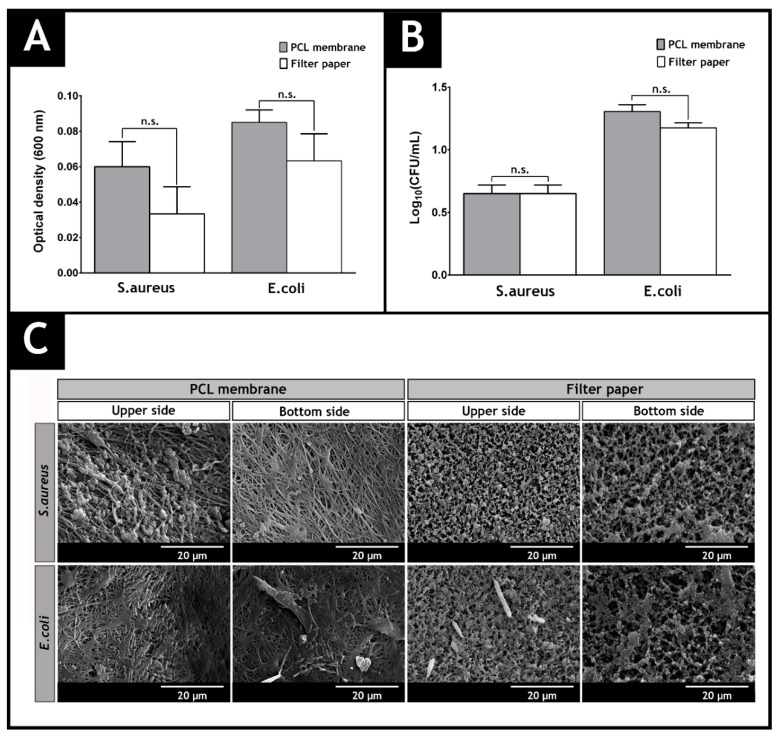
Evaluation of the bacterial infiltration through PCL membrane and filter paper (control group). The optical density of the medium culture present in the lower chamber of the transwell system determined at 600 nm is shown in (**A**); The number of colony forming units of *S. aureus* and *E. coli* that cross the PCL and filter paper, after 24 h, are displayed in (**B**); SEM images of adhered microorganisms (*S. aureus* and *E. coli*) at the upper and lower side of the PCL membrane and filter paper are observed in (**C**).

The results presented in Figure 9 show that the top layer of the produced membranes acts as protective barrier, avoiding the infiltration of the model bacteria. The data obtained for this layer does not show any significant statistical difference with the control group, where filter paper (with pore size of 0.22 µm) was used. SEM images also evidenced that few bacteria were able to adhere to the upper side of the top layer. Such results can be explained by the low porosity of the PCL layer, that hampers the entrance of microorganisms into the wound.

**Figure 10:**
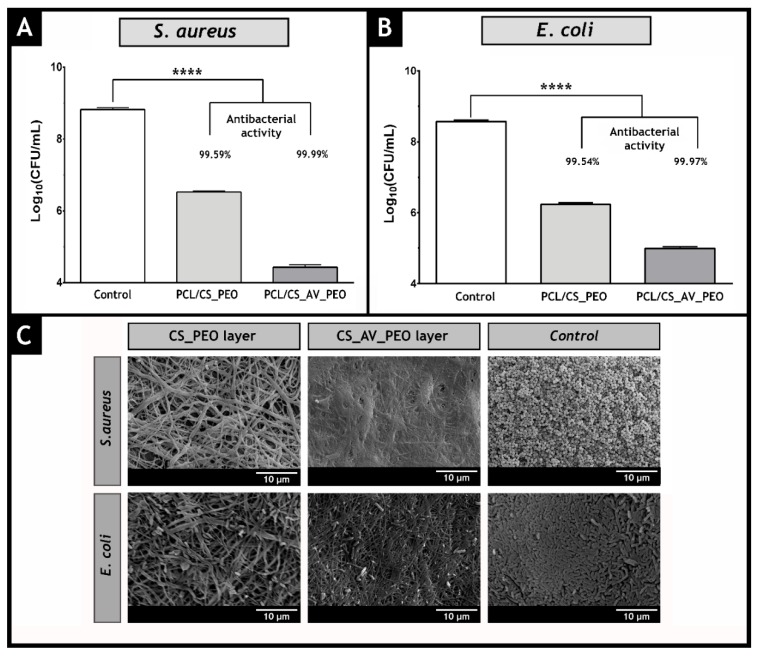
Evaluation of the bactericidal activity of the produced membranes. The antibacterial activity of PCL/CS_PEO and PCL/CS_AV_PEO membranes against *S. aureus* and *E. coli* is presented in (**A**,**B**), respectively; SEM images of *S. aureus* and *E. coli* in contact with PCL/CS_PEO and PCL/CS_AV_PEO membranes and of the negative controls are displayed in (**C**).

Additionally, the capacity of the bottom layers (CS_PEO and CS_AV_PEO) to provide an aseptic environment at the wound site was also evaluated. To accomplish this, membranes were incubated over 24 h with bacteria. Both bottom layers (CS_PEO and CS_AV_PEO) showed an inhibitory effect on *S. aureus* and *E. coli* growth (see Figure 10A,B). Furthermore, no biofilm formation on the membrane’s surface was noticed (see Figure 10C for further details) due to the intrinsic bactericidal activity of CS and AV.

In the literature, it has been described that this polycationic polymer interacts with the negatively charged groups present at the bacteria surface, leading to an increased cell wall permeability and consequently the leakage of intracellular constituents and the dissipation of the ionic gradients that exist in bacteria [21,115,116,117]. Furthermore, the positive charge density can be increased through a deacetylation process. Herein, the chitosan deacetylation degree was set to about 98% (see Table 1), i.e., almost all the primary amine groups of CS were positively charged and are available to interact with the negatively charged groups present in the bacterial cell wall. Additionally, the acemannan, anthraquinones, and salicylic acid present in AV gel also contribute to the bactericidal activity displayed by the produced membranes [35,118].

## 4. Conclusions

In this study, electrospinning technology was used to produce asymmetric membranes composed of PCL, CS, PEO and in some cases AV. The asymmetric structure of the membranes was conceived to mimic skin native structure. The top layer confers protection to the wound against external threats and, simultaneously, the bottom layer encourages cell migration and proliferation. The results attained show that the incorporation of AV in a CS_PEO nanofiber system enabled the production of a bottom layer capable of providing adequate moisture at the wound site as well as promoting better and faster fibroblast attachment and proliferation. In addition, the produced membranes presented mechanical properties and antimicrobial activity that are compatible with their applications as wound dressings.

In the near future, the incorporation of other bioactive molecules (e.g., ECM components, vitamins or growth factors) can be considered as a means to further improve the performance of these membranes in wound repair or other envisioned biomedical applications.

## Figures and Tables

**Table 1:** Degree of deacetylation of the different chitosan samples used in this study (*n* = 3).

Sample	Nominal DD ^a^ (%)	Determined DD ^b^ (%)
**(1) Comercial Chitosan**	75–85	82.02 ± 1.87
**(2) Purified Chitosan**	—	97.88 ± 1.19

^a^ Provided by the manufacturer; ^b^ Determined by first derivative UV-spectroscopy (1DUVS).
